# Design and operation of a molten salt electrochemical cell

**DOI:** 10.1016/j.mex.2022.101626

**Published:** 2022-02-01

**Authors:** Anthony N. Consiglio, Francesco Carotti, Ertai Liu, Haley Williams, Raluca O. Scarlat

**Affiliations:** a Department of Nuclear Engineering, University of California, Berkeley, CA 94720, United States; b Department of Engineering Physics, University of Wisconsin–Madison, Madison, WI 53706, United States

**Keywords:** FLiBe, Hydrogen, Voltammetry, High temperature, Gas control, CA, chronoamperometry, CE, counter electrode, CV, cyclic voltammetry, DRE, dynamic reference electrode, FHR, fluoride salt-cooled high temperature reactor, FLiBe, LiF-BeF_2_ (67–33 mol %), FLiNaK, LiF-NaF-KF (46.5–11.5–42 mol%), LSV, linear sweep voltammetry, MSR, molten salt reactor, RE, reference electrode, WE, working electrode

## Abstract

Molten salts such as 2LiF-BeF_2_ (FLiBe) have been proposed as coolants for advanced nuclear fission and fusion reactors. Critical to the design, licensing and operation of these reactors is characterization and understanding of the chemical behavior and mass transport of activation and fission products, corrosion products, and other solutes in the coolant. Electrochemical techniques are a powerful suite of tools for probing these phenomena. The design of an experimental cell for molten salt electrochemistry is described herein. As a demonstration of this design, details of the experimental methods used to conduct electrochemical experiments with molten FLiBe with addition of LiH are provided. Decommissioning of the cell is considered from the point of view of decontamination and waste generated. Main features of the cell include:

• Suitable for operation up to 800 °C; suitable for operation inside and outside of a glovebox.
• Enables sweep gas, gas sampling and analysis; enables addition of solid and liquid materials during operation.
• Supports a variety of electrode materials and arrangements.

## Graphical abstract

**Figure:**
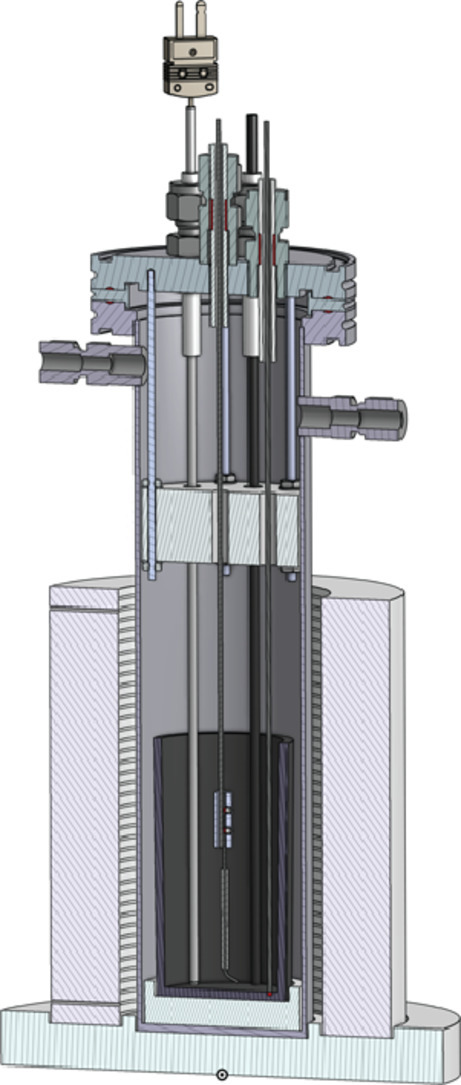

Specifications table

**Table:** 

Subject Area:	Chemical Engineering
More specific subject area:	Molten salt electrochemistry
Method name:	High-temperature molten salt electrochemical cell
Name and reference of original method:	N/A
Resource availability:	*Engineering drawings, list of materials, and 3D CAD models are available in the supplemental materials*

## Introduction

Molten salts have been proposed as a coolant for fluoride salt-cooled high temperature reactors (FHR), liquid fueled molten salt reactors (MSR) and fusion reactors [1], [2], [3]. The chemistry of these salts is critical to reactor safety in operation through corrosion of structural components, diffusion and solubility of corrosion, activation, and fission products, and through the processing of fuel. As an example, tritium, a radioactive isotope of hydrogen, is continuously produced in these reactors, and thus, understanding its chemical behavior and transport is of special interest. Electrochemical techniques were recently employed by Carotti et al. to study these phenomena in 2LiF-BeF2 (FLiBe)[4,5]. Described herein is the design of the electrochemical cell used to conduct these experiments, along with details of its operation.

Many electrochemical cell designs have been used for molten fluoride studies, but this cell was designed to benefit from a compact design, lack of water-cooling requirement, ease of electrode exchange, and possibility of gas-phase control and gas-phase monitoring. Most molten salt electrochemical cells are located within inert gas gloveboxes to avoid oxygen and moisture; therefore, a compact design is desired to conserve valuable glovebox space. This design uses a slim cylindrical heater rather than an insulated box furnace to minimize its footprint. To avoid over-heating of the top flange gasket and ensure a sealed cell, several studies use water cooling [6], [7], [8]. Alternatively, a heat shield, like those used by [9], is used in this design to accommodate temperatures up to 650 °C. Some cells use long electrodes which pass from outside the cell, through the top flange to the crucible of salt [10]. Others, like this design, use set screw connectors to join the electrode and a conducting extension rod which interfaces with the potentiostat [11]. This allows for shorter precious-metal electrodes and manipulation of the electrode materials without major adjustments to the top flange. Overall, this design provides basic functional requirements with extended ease of use and flexibility of operation.

## Electrochemical cell design

**Fig. 1:**
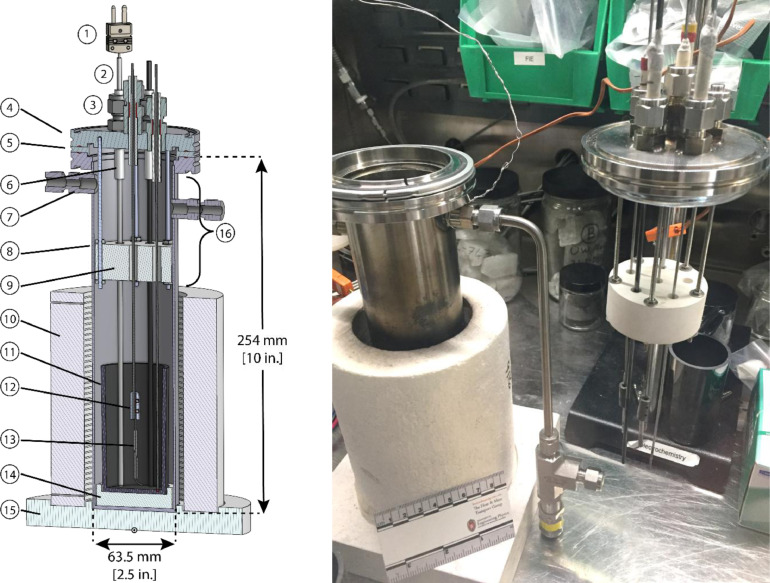
(a) Cross-section view of electrochemical cell. (1) Thermocouple, (2) thermowell, (3) Swagelok tube fittings (5x), (4) top lid, (5) centering ring, (6) alumina tube, (7) gas control tube fittings, (8) cell body, (9) heat shield, (10) furnace, (11) crucible, (12) electrode connector, (13) electrode sleeve, (14) centering disk, (15) furnace base, (16) active cooling region. (b) Photograph of electrochemical cell with top cell flange assembly removed from cell body.

Electrochemical experiments are performed using the experimental cell depicted in Fig. 1. Detailed engineering drawings, list of materials and 3D CAD models of the cell assembly and components are available in the supplemental materials.

The main body of cell consists of a 304 L stainless steel tube capped at the bottom and with an ISO K-style bored flange at the top (QF63-SWK, Kurt J. Lesker). The cell has an inner diameter of 60.2 mm (2.37″) and a nominal height of 254 mm (10″). The top lid is an ISO K-style blank flange (QF63-BK, Kurt J. Lesker) machined with six feedthroughs, onto which tube fittings are welded (SS-400–1-4WBT, Swagelok). A stainless-steel centering ring with fluorocarbon o-ring (QF63-SAVR, Kurt J. Lesker) is used to mate the top flange and cap.

The Swagelok fittings in the top flange allow for access into the cell for electrodes and an N-type thermocouple. To avoid interactions between the thermocouple and the fluoride molten salt, a 1/8 in. OD × 12 in. long closed-end molybdenum tube (3.175- × 304.8 mm) with an inner diameter of 2.4 mm (0.095 in), is used as a thermowell (Rhenium Alloys). The thermowell and electrodes are sealed around alumina tubes (Ortech, Inc., 6.35 mm OD x 3.2 mm ID x 70 mm Long (0.250″ x 0.125″ x 2.755″), and 6.35 mm OD x 1.6 mm ID x 70 mm Long (0.250″ x 0. 0625″ x 2.755″)) using a high-temperature ceramic adhesive (part no. 571, Aremco). The alumina tubes are then inserted through the Swagelok fittings and serve as high-temperature electrical insulators for the electrodes, preventing electrical contact with the fittings or flange. Teflon ferrules are used for Swagelok connectors to avoid damage of the alumina tubes if excessive torque is applied to the Swagelok nuts.

Two Swagelok feedthroughs (SS-400–1-4WBT, Swagelok) are welded to opposite sides of the main cell body and are intended as inlet and outlet gas ports for experiments where a controlled gas flow is required. In the experiments reported here, gas phase control was not required. To prevent over-pressurization of the cell, a pressure relief valve (SS-RL3S4, Swagelok) is connected to one of the side Swagelok ports.

The salt sample is contained within a glassy carbon crucible (GAT 13, HTW Germany) with a volume of 131 ml, height of 90 mm and opening diameter of 44 mm. The crucible is placed on top of a Macor support (Precision Ceramics USA), positioned at the bottom of the cell. The Macor support provides electrical isolation of the crucible from the electrochemical cell and centers the crucible in the cell body, avoiding shorting between the electrodes and the crucible.

A 700 W ceramic radiant heater (CRFC36/115A, Omega) is employed to melt the salt and achieve an elevated operating temperature of 500 °C-800 °C. The vertical cylindrical furnace, which surrounds the bottom portion of the cell, is 152.4 mm (6 in.) tall and has a bore diameter of 76.2 mm (3 in.). Two thermocouples are installed into the body of the furnace to monitor its temperature. The temperature of the salt is controlled by regulating the furnace power using a silicon-controlled rectifier (DIN-A-MITE® C, Watlow). In order to ensure stable thermal conditions in the salt and to reduce the temperature reached by components at the top of the cell, a 25.4 mm (1 in.) thick, 58.4 mm (2.3 mm) diameter boron nitride disk (AX05, Precision Ceramics) is positioned in the cell at an elevation just above the top of the furnace via five rods that thread into the underside of the tip lid. Six 6.4 mm (0.25 in.) diameter holes are machined into the block to enable passage of the thermocouple and electrodes. In practice, this setup allowed for temperature homogeneity in the salt to within <±5 °C [5].

The upper flange temperature must be maintained below 200 °C to preserve the integrity of the fluorocarbon gasket and the Teflon ferrules. Based on testing of the upper flange temperature, it is determined that heat shield alone permits operations of the cell up to 650 °C. Higher operating temperatures up to 800 °C are achievable with the implementation of active cooling around the upper portion of the cell, which the cell is designed to accommodate.

## Electrodes

Two 1 mm (0.04 in.) diameter platinum wires (PT005156, Goodfellow) are used as the working electrode (WE) and quasi-reference electrode (RE). A 3.2 mm (0.125 in.) diameter glassy carbon rod (Sigradur® rod, HTW) serves as a counter electrode (CE). In order to minimize the required length of the platinum conductors, 316 stainless steel connectors are used to join the platinum wires and 1/16 × 12in. (1.59 × 304.8 mm). molybdenum rods (01016740, H.C. Starck Inc.), which extend to the top of the cell. Two #2–56 × 1/8″ set screws per connector fix the electrodes in place.

**Fig. 2:**
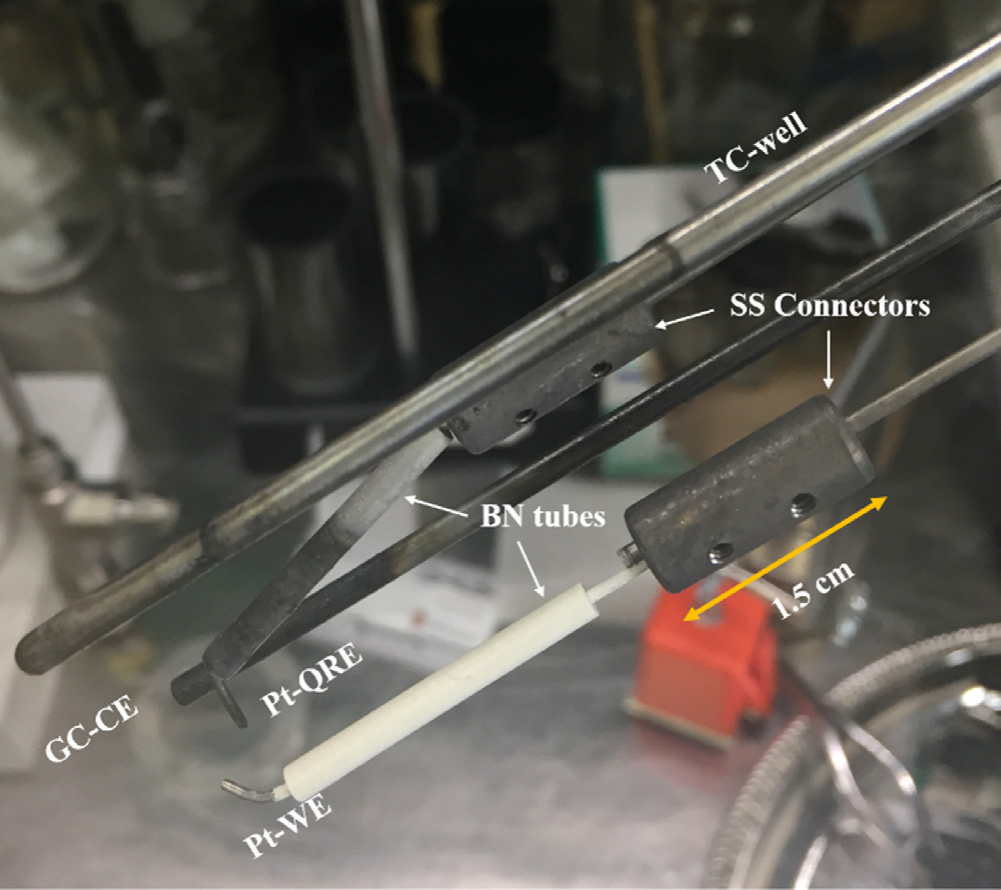
Electrode configuration consists of a glassy carbon counter electrode (CE), Pt working electrode (Pt-WE) and a quasi-reference electrode (Pt-RE). A Molybdenum thermowell (TC-well) protects thermocouple from direct contact with salt.

**Fig. 3:**
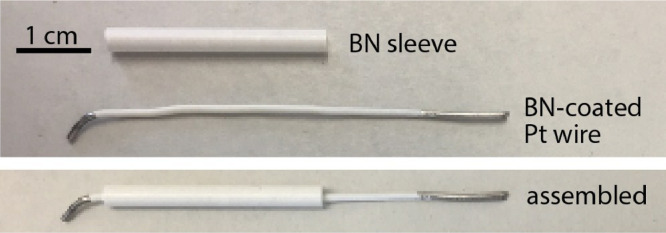
Pt electrode coated with BN aerosol spray and BN tube.

In the experiments reported by Carotti et al. [12], it is found that upon addition of the LiH to the melt, a floating conductive layer forms on the salt surface. To prevent this from causing shorting between electrodes, boron nitride tubes (BN 99, Stanford Advanced Materials, 3.175 mm OD x 1.588 mm ID x 30 mm long (0.125″ x 0.0625″ x 1.2″)) and boron nitride aerosol spray (Precision Ceramics) are applied around the Pt WE and Pt RE. This electrode shielding configuration effectively eliminated the shorting effect that was otherwise observed in the FLiBe + LiH experiments. The BN sprayed layer was also deemed useful to define the surface area of the Pt WE. To support the BN tubes and prevent them from slipping off the Pt wire, the WE and RE tips are bent at an approximate angle of 30° This geometry causes a non-optimal potential distribution between the electrodes that should be modified to increase signal quality (Fig. 2, Fig. 3).

In the experiments reported in [12], a quasi-RE is used in placed of a thermodynamic RE. Since quasi-REs do not provide a thermodynamic potential reference and the potential stability is predicated upon a stable chemical condition of the salt, the dynamic reference electrode (DRE) technique is performed before each experiment to allow the potential read by a quasi-RE to be placed on a thermodynamic scale [13]. By applying a current pulse, beryllium is reduced and electroplated onto the surface of the Pt-WE. The open circuit potential between the quasi-RE and the Pt-WE is then measured. This allows Pt quasi-RE voltages to be reported versus the Be/Be^2+^ redox couple.

The electrochemical cell design allows for different reference electrode configurations, and as such, an additional molybdenum electrode may be added to be used as WE for the DRE procedure. Preventing electroplating beryllium on the platinum wire simplifies the tests and increases the speed of the electrochemical measurements. The electrochemical cell is also designed to house a Ni/Ni(II) thermodynamic reference electrode, designed as described in detail in[14,15].

## Testing environment

**Fig. 4:**
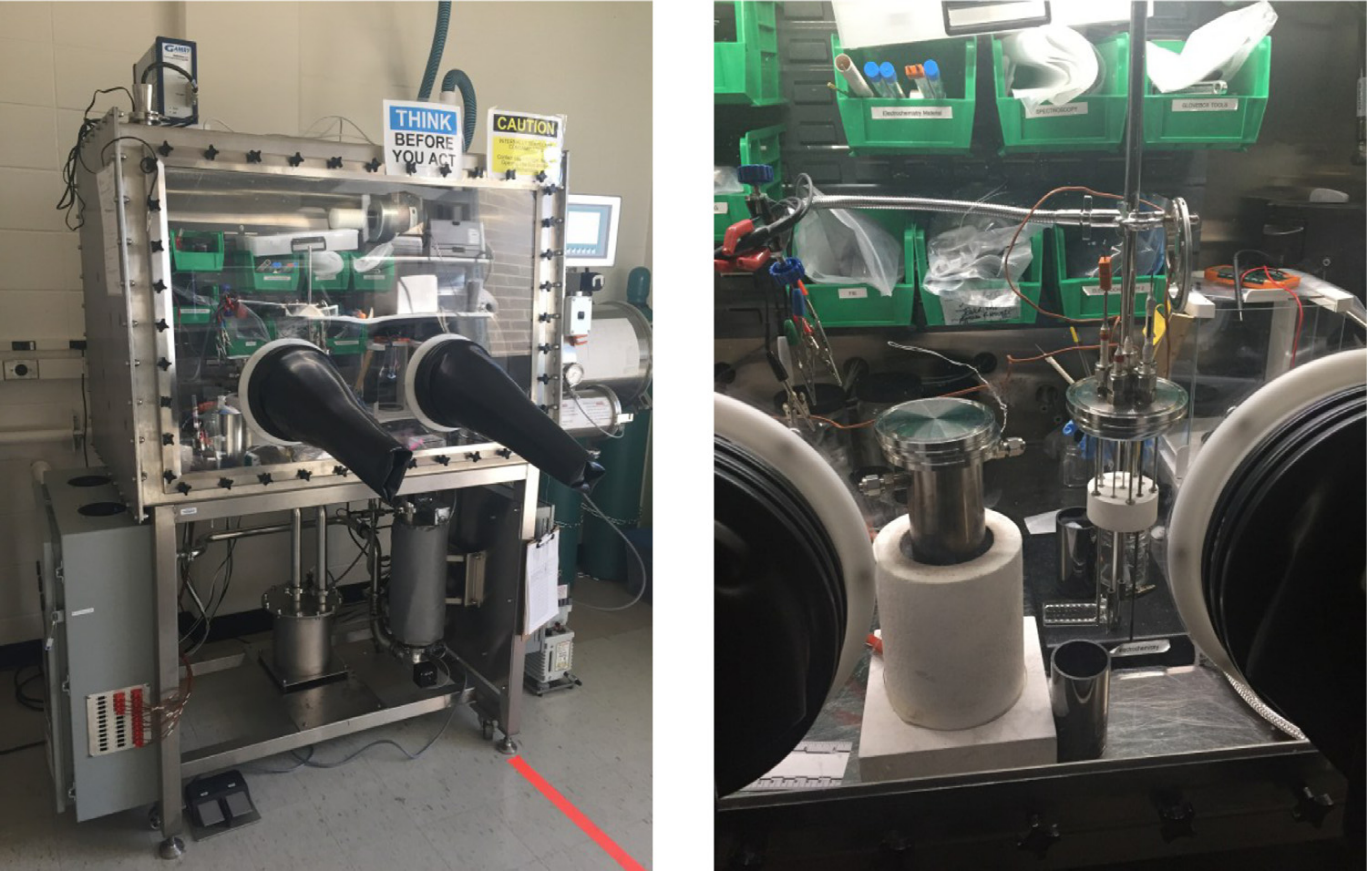
(a) Argon glovebox (LC Technology Solutions, Inc.). (b) Electrochemical cell in glovebox.

All work is conducted in an argon glove box (LC Technology Solutions Inc.), as shown in Fig. 4. Electrochemical measurements are performed using a Gamry Reference 600 potentiostat kept outside the glovebox (see Fig. 4). The potentiostat cable is embedded in a vacuum-rated feedthrough mounted on a KF40 flange to enter the glovebox environment (LDS Vacuum). The atmosphere in the glovebox is maintained at a moisture and oxygen level below 1 ppm. The glovebox ensures that the electrochemical testing is performed in inert environment. To avoid damaging electronics located inside the glovebox, the atmospheric temperature inside the glovebox is continuously monitored, and if the temperature rises above 40 °C, the furnace is automatically turned off.

In the case that neither containment nor gas phase control is required, the electrochemical cell may also be operated in a fume-hood. A separate instance of the electrochemical cell described here has also been successfully operated in a fume-hood for electrochemical corrosion studies in LiF-NaF-KF (FLiNaK) salt [11].

## Details of experimental procedure used in [12]

The experiments presented in [12] investigate the diffusivity of hydrogen in FLiBe using the electrochemical cell to conduct linear sweep voltammetry (LSV), cyclic voltammetry (CV) and chronoamperometry (CA) measurements. The procedure employed in these studies is detailed below.

### Electrode preparation and cleaning

• Before first use, the materials to be used in the electrochemical cell are cleaned using ethanol and laboratory residue-free wipes. Special attention shall be paid to materials that will be in contact with the salt (electrodes and thermowell).
• Application of BN aerosol spray to the Pt wires:
  ○ Cover with tape the area of the electrodes that should remain bare.
  ○ Apply the BN spray. For maximum adherence, very thin layers should be applied to the electrode in multiple coats, allowing several minutes of drying time between applications.
  ○ Remove the tape and inspect the quality of the BN deposited. The coating should appear thin and smooth, with no cracks or imperfections.
  ○ Slide the BN tube on the electrode to further protect against electrical shorting due to the possible presence of a floating conductive film on the melt surface.
  ○ Note: In the experiments reported in [12], the BN layer did not show signs of deterioration for the time that it was immersed in highly reduced FLiBe (FLiBe + LiH) at 600 ˚C (the longest test lasted over 36 h). Further qualification is required to establish long term stability of this layer.
• Electrode cleaning between experiments:
  ○ It has been observed that inserting and removing the electrodes through the floating conductive layer on the salt surface caused a film of the conductive material to deposit on top of the BN protective coats and tube, which ultimately caused shorting to occur.
  ○ If shorting cannot be avoided, the WE and RE should be removed and cleaned as follows:
    - Withdraw Pt WE and RE from cell and remove BN sleeves.
    - Clean Pt wires in ultrasonic bath with deionized (DI) water and with nitric acid to remove salt deposits, impurities deposits and BN coating.
    - Polish electrode surface with 1200-grit sandpaper.
    - Re-apply BN layer and BN sleeves as detailed above. In the case that the BN sleeve becomes too tarnished, a new BN sleeve may be used.

### Set up and blank electrochemical measurements

• Recommended initial step (not used in the study reported in [12]): Grind bulk FLiBe samples with mortar and pestle to ensure homogeneity of constituents.
• Weigh desired quantity of FLiBe.
• Place FLiBe in glassy carbon crucible and place crucible the cell.
• Assemble the cell top lid with electrodes and thermowell:
  ○ Install heat shield on underside of lid.
  ○ Adjust electrode heights based on salt volume and desired immersion level.
  ○ Verify electrode proper installation and geometry by inserting into empty cell.
  ○ Test for electrical shorting between electrodes and crucible.
• Before melting the salt, place a blank flange or lid on cell.
• Turn on furnace power and control system.
• Adjust furnace to raise the temperature to the desired level above FLiBe melting temperature.
• Once cell has stabilized at desired temperature and FLiBe is melted, remove blank flange, and install centering ring.
• Install the top lid with heat shield, electrodes, and thermocouple on the cell.
• Perform dynamic reference electrode (DRE) procedure to evaluate the potential of the Pt-RE vs Be/Be^2+^ [13].
• Perform LSV, CV and CA measurements in FLiBe without LiH to form baseline datasets.

### While molten salts are good ionic conductors, resistances in the range of 1Ω may be expected [5]. Thus, the ohmic drop should be assessed before each experiment. LiH addition to the molten FLiBe

• Weigh desired quantity of LiH using weighing paper.
• Disconnect potentiostat cable and remove the cell top lid (see Fig. 1b).
• Insert glass funnel into cell.
• Add weighed quantity of LiH to melt using glass funnel.
• Remove the funnel from the cell and allow time for cooldown.
• Re-insert the cell top lid assembly.
• Note: To confirm the amount of reagent added to the salt, the funnel and weighing paper are weighed before and after use to determine the amount of residual powder left behind.

### Electrochemical measurements

The following measurements are performed using the three-electrode setup: Pt WE, Pt RE, glassy carbon CE.

• Conduct dynamic reference electrode (DRE) procedure to evaluate the potential of the Pt-RE vs Be/Be^2+^ [13].
• Perform LSV measurements to determine potential where oxidation of hydrogen occurs. Oxidation is identified by peaks in the current response compared to the baseline measurement.
• Once the bounds of the oxidation window are determined, perform CV scans.
  ○ The reversibility of the electrochemical reaction can be assessed by comparing the magnitude of the cathodic and anodic current peaks. A reversible reaction is characterized by a ratio of anodic to cathodic peak current close to unity.
  ○ Vary CV scan rate to investigate effect on reversibility and current response.
    - If greater reversibility is observed for faster scan rates, an irreversible reaction that consumes the product of the electrochemical reaction may be present. The method developed by Nicholson and Shain [16] can be used to assess whether this mechanism is present and to determine the reaction rate for the irreversible reaction.
  ○ Estimate the number of electrons exchanged recognizing that the difference in potential (V) between cathodic and anodic current peaks for a reversible reaction is related by the Nernst equation as ΔE=2.3RT/nF where R is the ideal gas constant in J/mol/K, T is the temperature in K, n is the number of electrons exchanged, F is Faraday's constant in C/mol.
• Perform LSV measurements at various scan rates.
  ○ A relation for the diffusion coefficient and concentration for reversible reactions with soluble redox species can be obtained using the Randles-Sevcik equation (other equations can be applied for nonsoluble species, or non-reversible electrochemical reactions):
    (1) $$\;i_{p}=0.4463{\left(\frac{F^{3}n^{3}D\nu }{RT}\right)}^{1/2}AC^{*}$$
    where $i_{p}$ is the LSV peak current in A, ν is the scan rate in V/s, D is the diffusion coefficient in cm^2^/s, $C^{*}$ is the concentration of $H_{2}$ in mol/cm^3^, F is Faraday's constant in C/mol, n is the number of electrons exchanged, R is the ideal gas constant in J/mol/K, T is the temperature in K, and A is the active area of the working electrode in cm^2^.

• Perform CA measurements by applying a potential step and recording the current response over time.
  ○ It is important that the potential is stepped from a potential where hydrogen (i.e. species of interest) is not oxidized (or reduced) to a potential where the current is diffusion controlled.
• A second relation between the diffusion coefficient and concentration is obtained using the Cottrell equation, which predicts the transient current response, i(t).
  (2) $$\;i\left(t\right)=\frac{nFAD^{1/2}C^{*}}{\pi^{1/2}t^{1/2}}$$
  where the current i(t) is in A, n is the number of electrons exchanged, F is Faraday's constant in C/mol, A is the area of the working electrode in cm^2^, D is the diffusion coefficient in cm^2^/s, C is the concentration of H_2_ in mol/cm^3^, and t is time in s.

• Under the framework of the Randles-Sevcik and Cottrell models, the diffusion coefficient or concentration of hydrogen can be computed, if one or the other is known.
• For each LSV, CV, or CA scans, the electrode may be pre-equilibrated to the potential window to be scanned. This must be specified for each technique, along with potential window limits, scan rate, etc.

## System cooldown

• Turn off power to furnace.
• Disconnect electrodes from potentiostat.
• Before salt freezes, remove top lid assembly with electrodes, thermocouple, and heat shield.
• Purge glovebox to dilute any gaseous products that may be released from cell.
• Allow the open cell to cool down.

## Decommissioning and disposal

Since the cell may be used with hazardous chemicals such as beryllium or with radioactive isotopes such as uranium or thorium, it may become contaminated and require special disposal. The modular construction of the cell enables easy disassembly into smaller, manageable components. The heat shield and electrodes can be completely disassembled into individual components. While components of porous or complex geometry may need to be disposed of as contaminated waste, other components can be decontaminated, limiting the amount of contaminated waste generated. The main cell body, as well as the top lid with access ports, are both single weldments. Approximate weights for the electrochemical cell and major components are as follows:

• Full cell assembly excluding furnace and furnace base: 2.7 kg (6 lbs.).
• Cell body: 1.1 kg (2.5 lbs.).
• Top lid: 0.8 kg (1.7 lbs.).
• Heat shield block: 0.2 kg (0.5 lbs.).
• Crucible: 0.07 kg (0.15 lbs.).

## Outlook

The electrochemical cell presented herein is designed for experiments at temperatures up to 800 °C and is specifically well suited for investigations of solubility, diffusivity, and reactivity of gases and other species dissolved in molten salts. In the experiments conducted by Carotti et al. [12], LiH serves as a hydrogen donor when added to FLiBe and was chosen as a first test in conducting experiments with this cell. These experiments were performed in an argon gas glovebox; however, the cell may also be operated in a fume hood.

The design of the electrochemical cell enables customization and interchanging of electrodes, and, through integration of gas sampling ports, enables continuous monitoring and analysis of the gas phase in the cell using a gas-chromatograph mass spectrometer, or other similar instrument. This capability may also be combined with the use of gas sparging, to perform gas-salt studies. Additionally, the cell is suitable for corrosion studies accompanied by electrochemical measurements, and for a whole suite of electroanalytical techniques.

## Co-submission

Co-submission with current paper to Electrochimica Acta.

Supplementary material

Provided along with this manuscript as supplemental material are a detailed engineering drawing of the cell assembly with bill of materials and 3D CAD files (*.STEP) of the cell assembly and components.

## Declaration of Competing Interest

The authors declare that they have no known competing financial interests or personal relationships that could have appeared to influence the work reported in this paper.

## Appendix Supplementary materials

Image, application 1

Image, application 2
