# Supplementary material for: Design and operation of a molten salt electrochemical cell
Source: MethodsX. 2022 Feb 1;9:101626. doi: 10.1016/j.mex.2022.101626 (PMC8892162; doi:10.1016/j.mex.2022.101626)
Supplement: Supplementary file 2 [file mmc2.pdf]

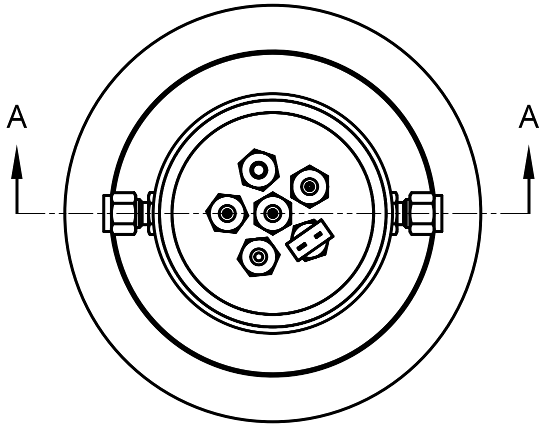

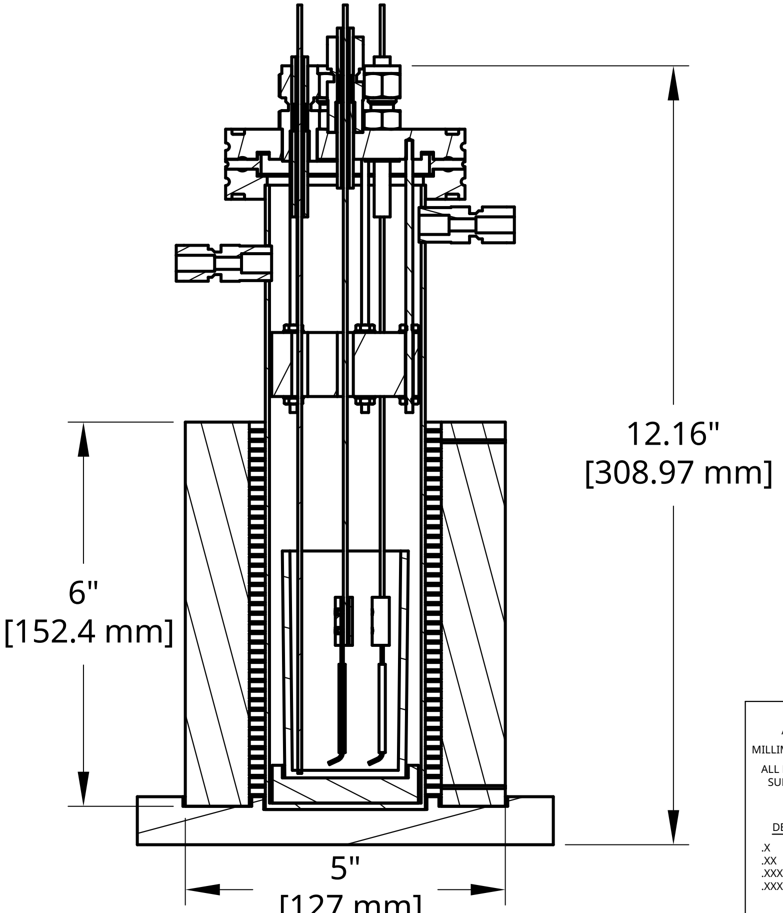

SECTION A - A

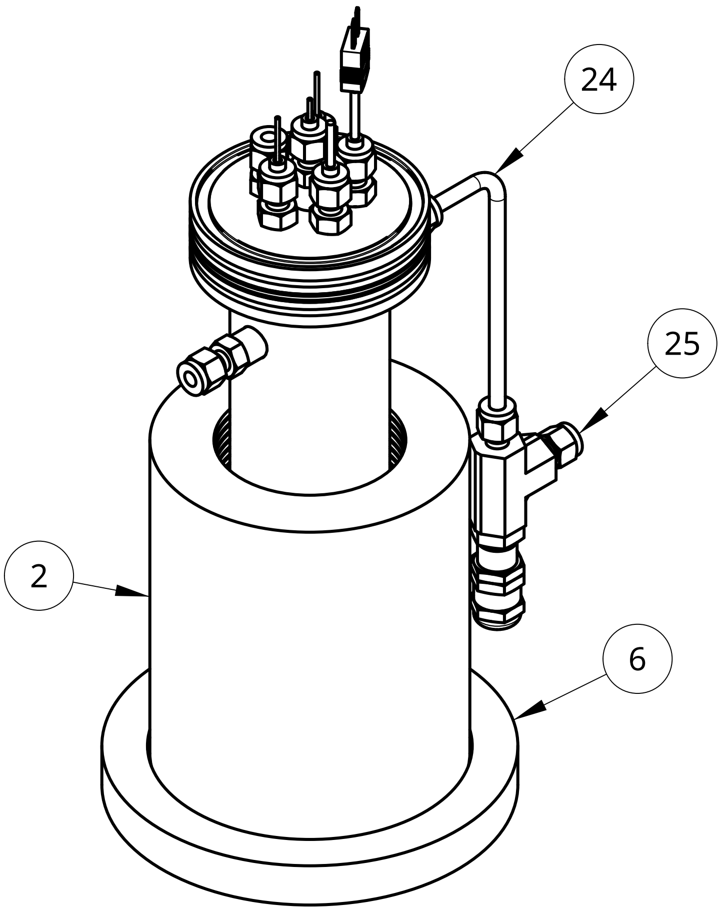

UNLESS NOTED OTHERWISE		NAME	DATE
ALL DIMENSIONS ARE IN INCHES	PREPARED		
MILLIMETER DIMENSIONS ARE SHOWN IN [ ]	REVIEWED		
ALL MACHINED SURFACES SHALL HAVE A SURFACE FINISH OF R250A OR BETTER	APPROVED		
TOLERANCES			
DECIMAL: .X ± 0.1; .XX ± 0.01; .XXX ± 0.005; .XXXX ± 0.0005			
FRACTIONAL: 0" TO 2" ± 1/16"; >2" TO 36" ± 1/8"; ANGULAR ± 1°			
DO NOT SCALE DRAWING	PROJECT		
BREAK ALL SHARP EDGES AND REMOVE BURRS	UCBS-07		
THIRD ANGLE PROJECTION			

THE SALT RESEARCH GROUP
Berkeley UNIVERSITY OF CALIFORNIA Nuclear Engineering

TITLE			
ELECTROCHEMICAL CELL			
DWG NO. 07EG_002			REV. 0
SCALE 1:3	SIZE A	SHEET 1 of 7	

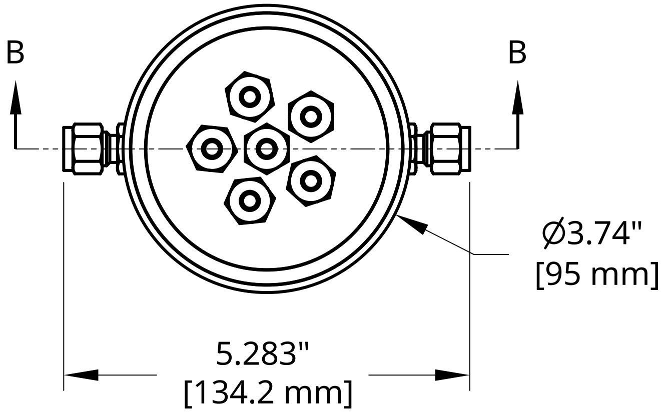

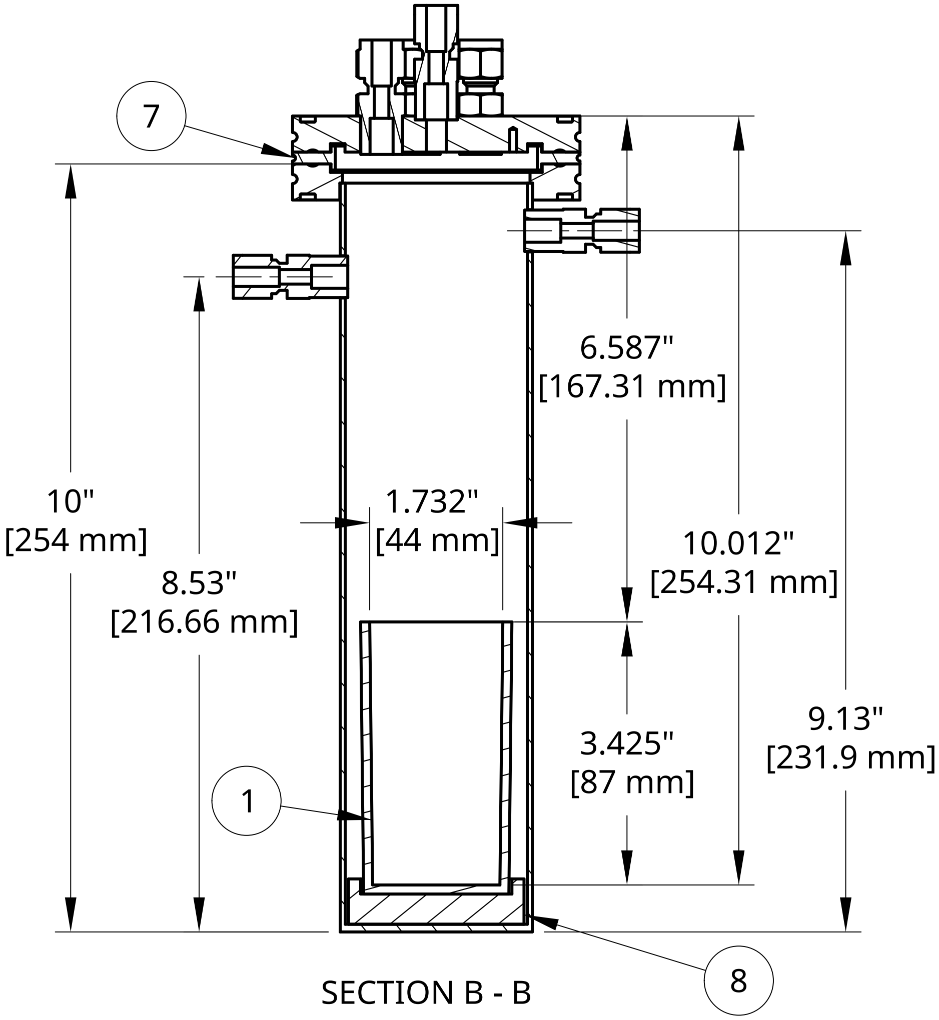

SECTION B - B

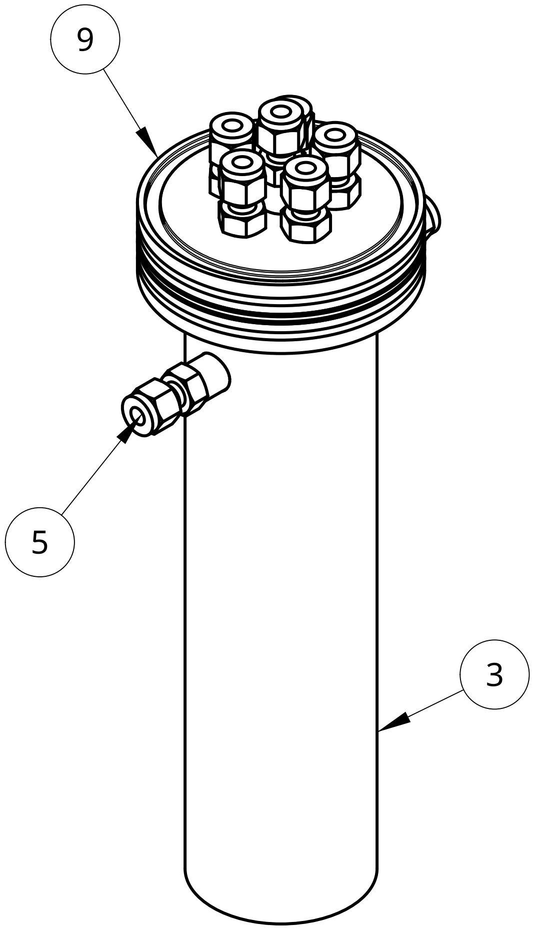

UNLESS NOTED OTHERWISE

ALL DIMENSIONS ARE IN INCHES
MILLIMETER DIMENSIONS ARE SHOWN IN [ ]
ALL MACHINED SURFACES SHALL HAVE A SURFACE FINISH OF R250A OR BETTER

TOLERANCES

DECIMAL		FRACTIONAL	
.X	± 0.1	0" TO 2"	± 1/16"
.XX	± 0.01	>2" TO 36"	± 1/8"
.XXX	± 0.005		
.XXXX	± 0.0005	ANGULAR	± 1°

DO NOT SCALE DRAWING
BREAK ALL SHARP EDGES AND REMOVE BURRS

THIRD ANGLE PROJECTION

PROJECT: UCBS-07

THE SALT RESEARCH GROUP
Berkeley UNIVERSITY OF CALIFORNIA Nuclear Engineering

TITLE: ELECTROCHEMICAL CELL

DWG NO.: 07EG_002

REV.: 0

SCALE: 2:5 | SIZE: A | SHEET: 2 of 7

Top Lid Assembly: Top lid, electrodes, heat shield, thermocouple

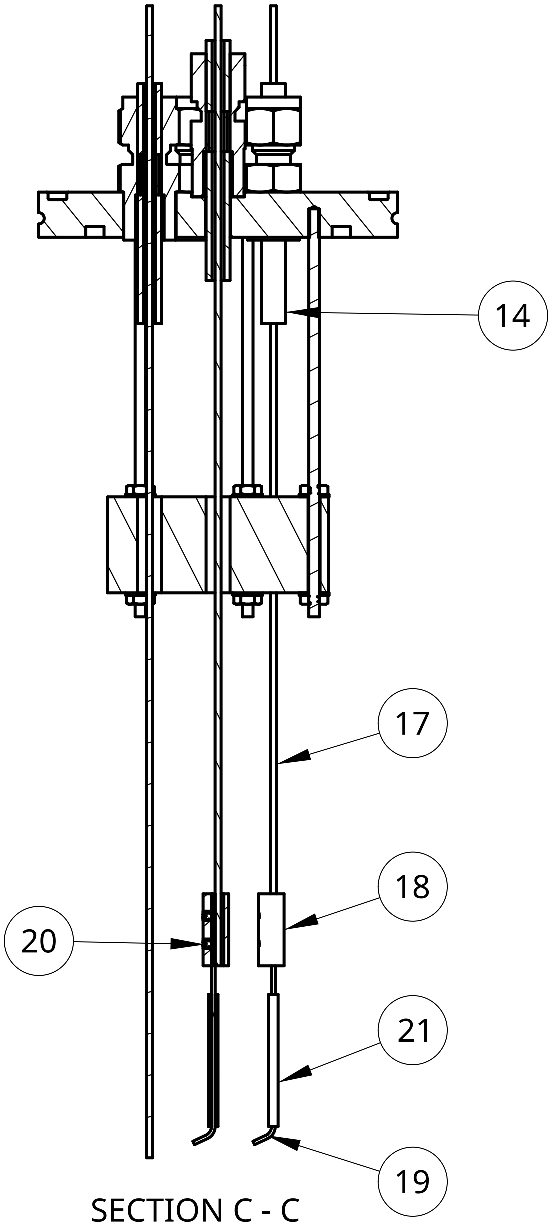

SECTION C - C

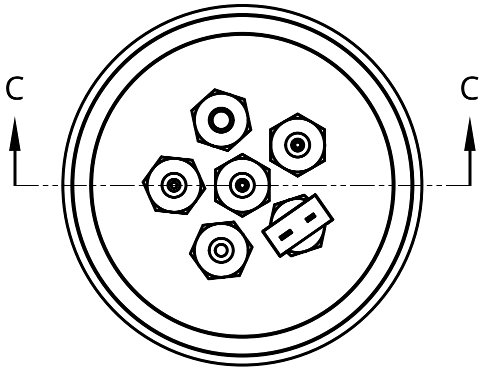

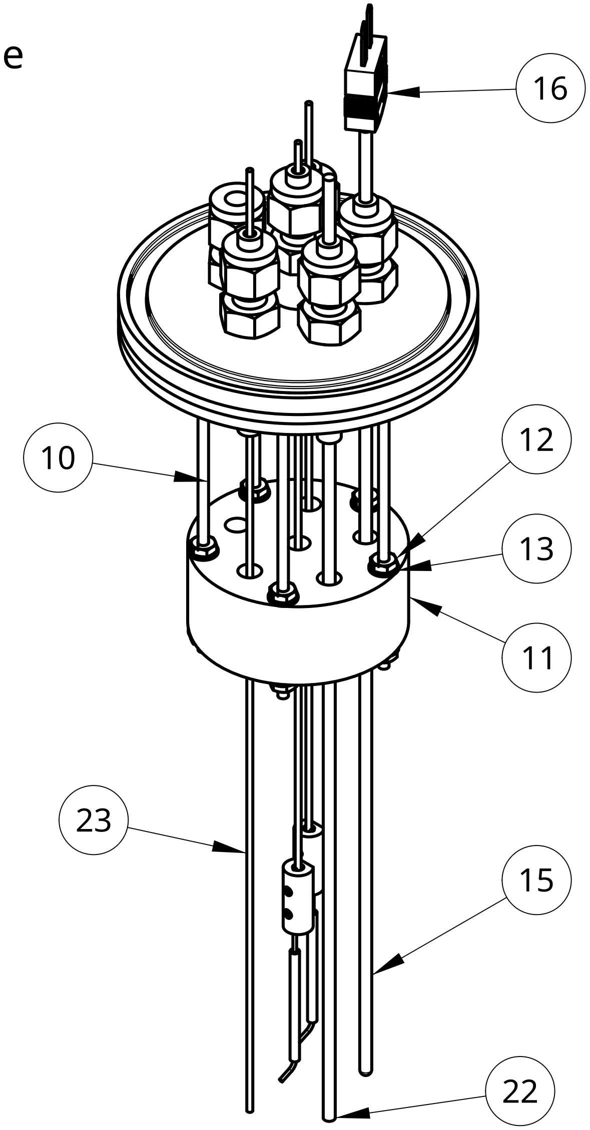

UNLESS NOTED OTHERWISE

ALL DIMENSIONS ARE IN INCHES
MILLIMETER DIMENSIONS ARE SHOWN IN [ ]
ALL MACHINED SURFACES SHALL HAVE A SURFACE FINISH OF R250A OR BETTER

TOLERANCES

DECIMAL		FRACTIONAL	
.X	± 0.1	0" TO 2"	± 1/16"
.XX	± 0.01	>2" TO 36"	± 1/8"
.XXX	± 0.005		
.XXXX	± 0.0005	ANGULAR	± 1°

DO NOT SCALE DRAWING
BREAK ALL SHARP EDGES AND REMOVE BURRS

THIRD ANGLE PROJECTION

PROJECT: UCBS-07

THE SALT RESEARCH GROUP
Berkeley UNIVERSITY OF CALIFORNIA Nuclear Engineering

TITLE: ELECTROCHEMICAL CELL

DWG NO.: 07EG_002

REV.: 0

SCALE: 1:2

SIZE: A

SHEET: 3 of 7

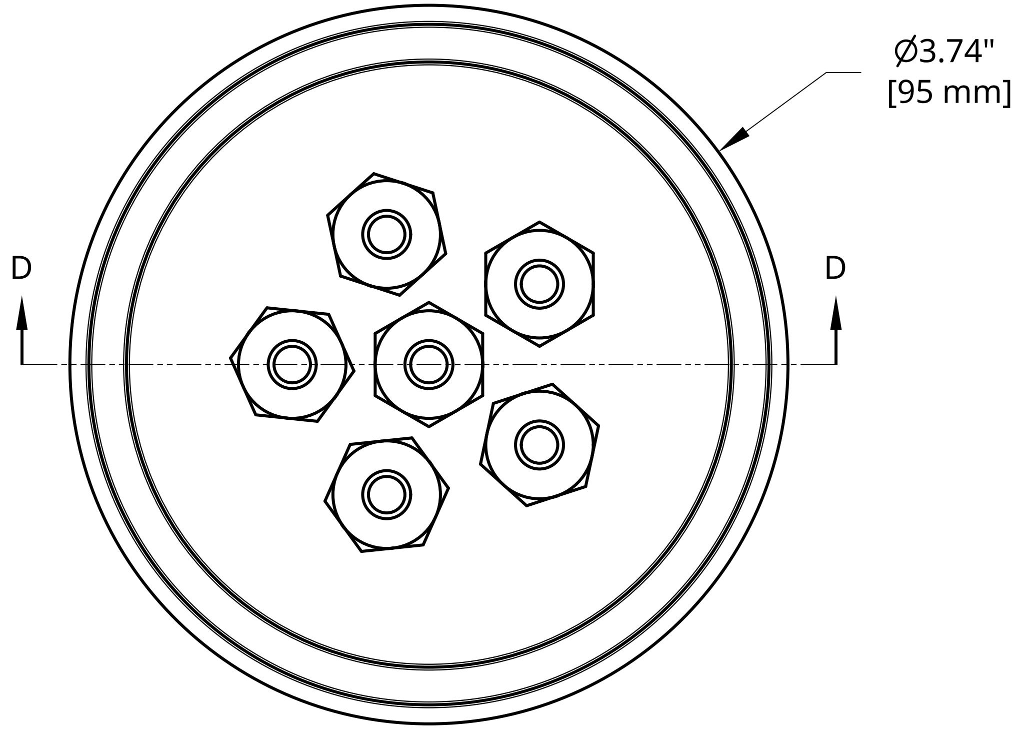

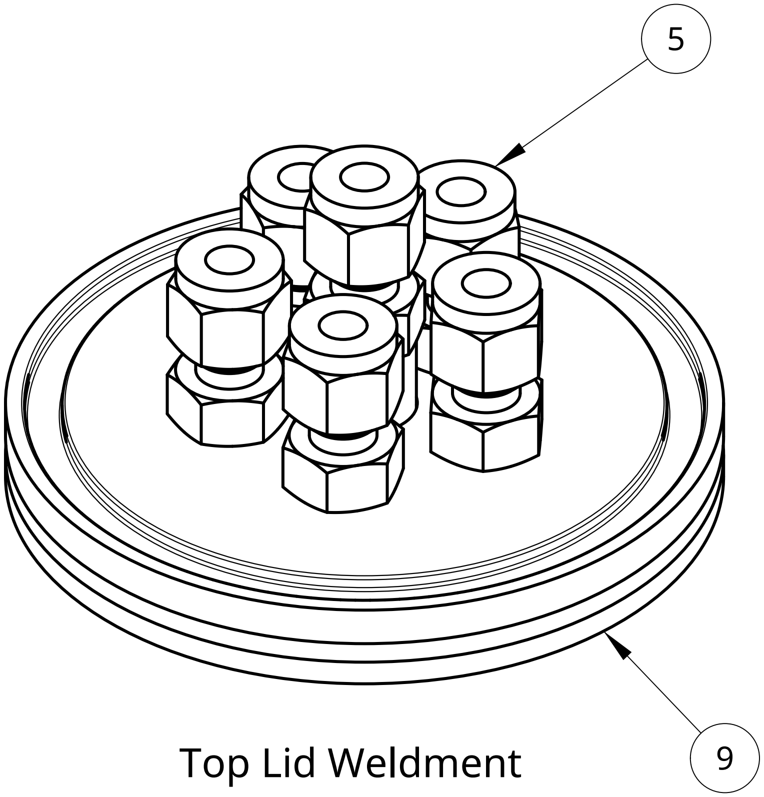

Top Lid Weldment

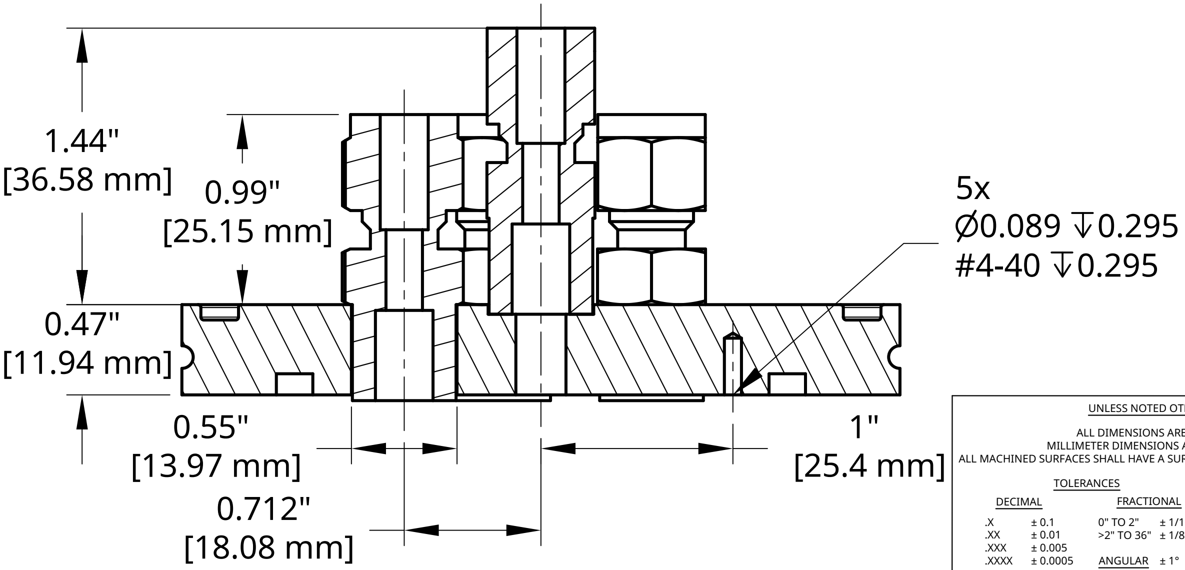

SECTION D - D

UNLESS NOTED OTHERWISE

ALL DIMENSIONS ARE IN INCHES
MILLIMETER DIMENSIONS ARE SHOWN IN [ ]
ALL MACHINED SURFACES SHALL HAVE A SURFACE FINISH OF R250A OR BETTER

TOLERANCES

DECIMAL		FRACTIONAL	
.X	± 0.1	0" TO 2"	± 1/16"
.XX	± 0.01	>2" TO 36"	± 1/8"
.XXX	± 0.005		
.XXXX	± 0.0005	ANGULAR	± 1°

DO NOT SCALE DRAWING
BREAK ALL SHARP EDGES AND REMOVE BURRS

THIRD ANGLE PROJECTION

PROJECT: UCBS-07

THE SALT RESEARCH GROUP
Berkeley UNIVERSITY OF CALIFORNIA Nuclear Engineering

TITLE: ELECTROCHEMICAL CELL

DWG NO.: 07EG_002

REV.: 0

SCALE: 1:1 | SIZE: A | SHEET: 4 of 7

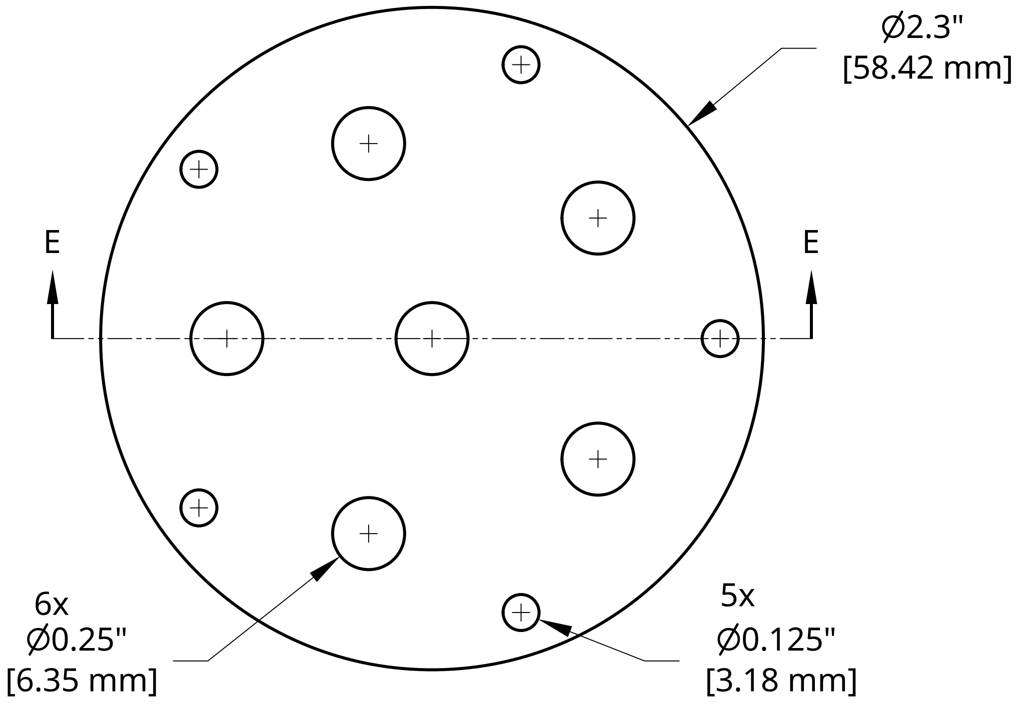

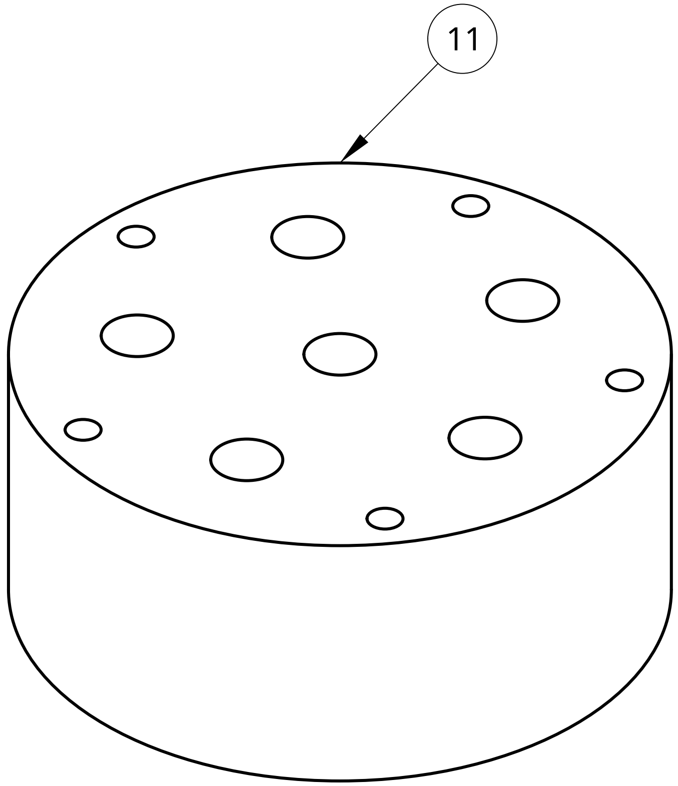

Heat Shield Block

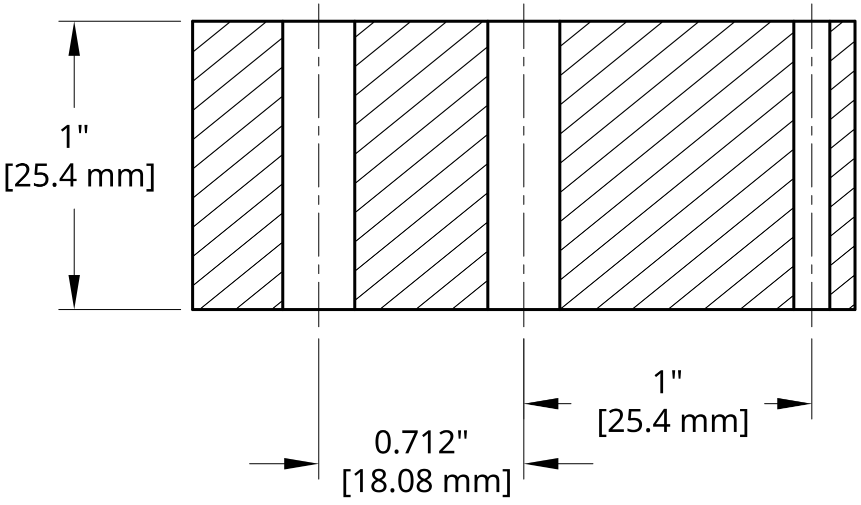

SECTION E - E

UNLESS NOTED OTHERWISE

ALL DIMENSIONS ARE IN INCHES
MILLIMETER DIMENSIONS ARE SHOWN IN [ ]
ALL MACHINED SURFACES SHALL HAVE A SURFACE FINISH OF R250A OR BETTER

TOLERANCES

DECIMAL		FRACTIONAL	
.X	± 0.1	0" TO 2"	± 1/16"
.XX	± 0.01	>2" TO 36"	± 1/8"
.XXX	± 0.005	ANGULAR	± 1°
.XXXX	± 0.0005		

DO NOT SCALE DRAWING
BREAK ALL SHARP EDGES AND REMOVE BURRS

THIRD ANGLE PROJECTION

PROJECT: UCBS-07

THE SALT RESEARCH GROUP
Berkeley UNIVERSITY OF CALIFORNIA Nuclear Engineering

TITLE: ELECTROCHEMICAL CELL

DWG NO.: 07EG_002

REV.: 0

SCALE: 3:2 | SIZE: A | SHEET: 5 of 7

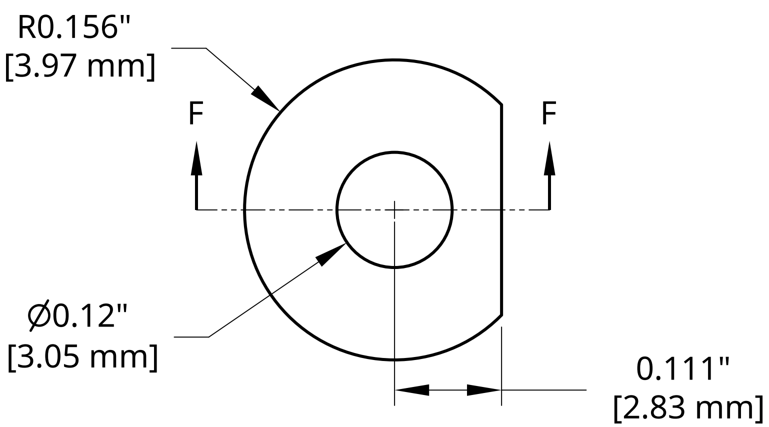

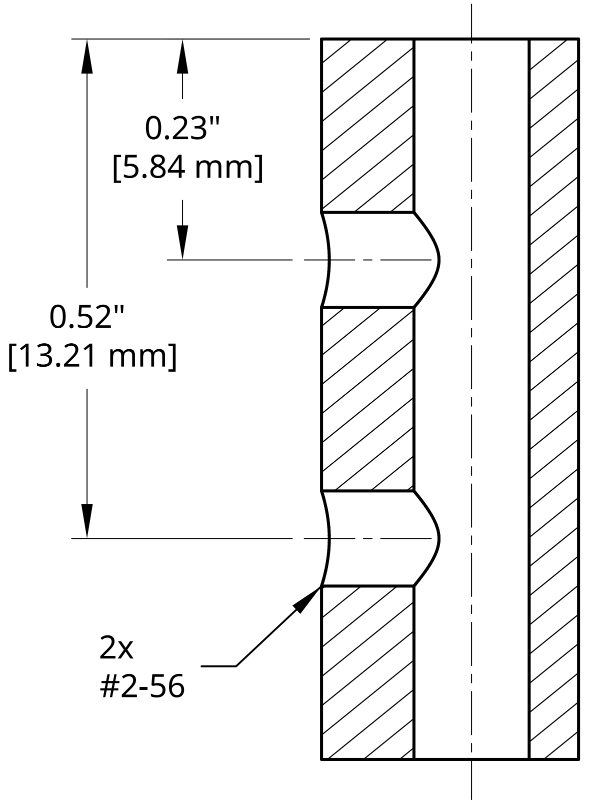

SECTION F - F

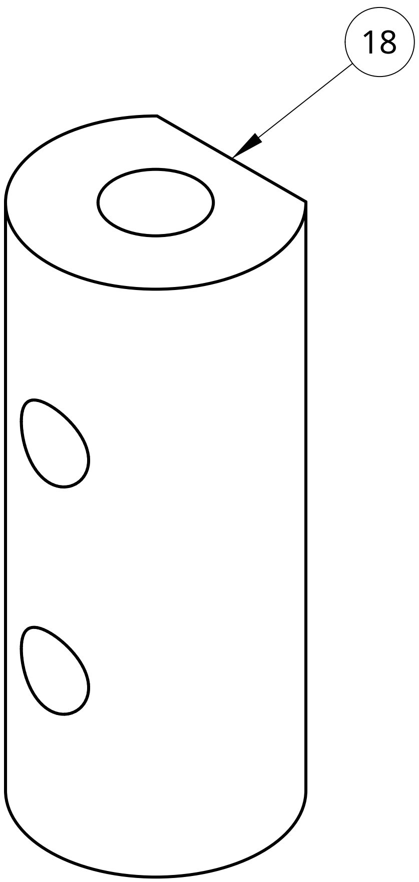

Electrode connector

UNLESS NOTED OTHERWISE

ALL DIMENSIONS ARE IN INCHES
MILLIMETER DIMENSIONS ARE SHOWN IN [ ]
ALL MACHINED SURFACES SHALL HAVE A SURFACE FINISH OF R250A OR BETTER

TOLERANCES

DECIMAL		FRACTIONAL	
.X	± 0.1	0" TO 2"	± 1/16"
.XX	± 0.01	>2" TO 36"	± 1/8"
.XXX	± 0.005		
.XXXX	± 0.0005	ANGULAR	± 1°

DO NOT SCALE DRAWING
BREAK ALL SHARP EDGES AND REMOVE BURRS

THIRD ANGLE PROJECTION

PROJECT: UCBS-07

THE SALT RESEARCH GROUP
Berkeley UNIVERSITY OF CALIFORNIA Nuclear Engineering

TITLE: ELECTROCHEMICAL CELL

DWG NO. 07EG_002 | REV. 0

SCALE 5:1 | SIZE A | SHEET 6 of 7

6 5 4 3 2 1

Item No.	Quantity	Part number	Name	Description	Material	Vendor
1	1	GAT 13	Crucible	Glassy carbon crucible	Glassy Carbon	HTW Germany
2	1	CRFC-36/115-A	Furnace	Omega CRFC Series Radiant Heater (700 W)		Omega
3	1	07EG_004	Cell Body	Tubing and Cap, SS304L, 2-1/2"OD, 0.065" Wall, ASTM A270 Polished OD & ID	SS-304	Kurt J. Lesker
4	1	QF63-SWK	Flange	Flange, ISO, SS, QF63 2.51" C-Bore, K-Style	SS-304	Kurt J. Lesker
5	8	SS-400-1-4WBT	Tube Fitting	Swagelok Tube Fitting, Bored-Through Male Connector, 1/4 in. Tube OD x 1/4 in. Male Pipe Weld	SS-316	Swagelok
6	1		Furnace Base		Alumina	
7	1	QF63-SAVR	Centering Ring	ISO Centering Ring (304 SS Ring, Fluorocarbon O-Ring)		Kurt J. Lesker
8	1		Crucible Base	Insulating base for crucible	Macor	Precision Ceramics USA
9	1	QF63-BK	Top Flange	Machined ISO-K Blank Flange (304L SS)	SS-304	Kurt J. Lesker
10	5	90575A148	Threaded Rod	Threaded Rod, 4-40 Thread Size, 4.25" Lg.	SS-316	McMaster-Carr
11	1	07EG_001_002	Heat Shield	Boron nitride heat shield block, 1" thick, 2.3" OD	Boron Nitride	Precision Ceramics USA
12	10	90257A005	Hex Nut	Hex Nut, 4-40 Thread Size	SS-316	McMaster-Carr
13	10	90107A005	Washer	Washer for #4 Screw, 0.125" ID, 0.312" OD	SS-316	McMaster-Carr
14	5		Alumina Tube	Alumina tube (0.25" OD, 0.125" ID, 2.5" Lg.)	Alumina	Ortech
15	1		Thermowell	Thermowell (0.125" OD, 0.015" wall thickness, 12" long)	Molybdenum	Rhenium Alloys
16	1		Thermocouple	Thermocouple		
17	2		Rod	Molybdenum rod (0.0625" OD, 10" long)	Molybdenum	H.C. Stark Inc.
18	2	07EG_003	Electrode Connector	Machined electrode connector block	SS-316	
19	2	PT005156	Electrode	Platinum electrode (1 mm diameter)	Platinum	Goodfellow
20	4	92313A016	Set Screw	2-56 Set Screw, 1/8" Lg.	SS-316	McMaster-Carr
21	2		Sleeve	Boron nitride electrode sleeve	Boron Nitride	
22	1		Electrode	Glassy carbon electrode (0.125" diameter, 12" length)	Glassy Carbon	HTW - Germany
23	1		Electrode	Molybdenum electrode (0.0625" OD, 12" long)	Molybdenum	H.C. Stark Inc.
24	1		Tube	1/4" Tube		
25	1	SS-RL3S4	Pressure Relief Valve	Stainless Steel Low Pressure Proportional Relief Valve (225 PSIG)	SS-316	Swagelok

UNLESS NOTED OTHERWISE

ALL DIMENSIONS ARE IN INCHES
MILLIMETER DIMENSIONS ARE SHOWN IN [ ]
ALL MACHINED SURFACES SHALL HAVE A SURFACE FINISH OF R250A OR BETTER

TOLERANCES

DECIMAL		FRACTIONAL	
.X	± 0.1	0" TO 2"	± 1/16"
.XX	± 0.01	>2" TO 36"	± 1/8"
.XXX	± 0.005	ANGULAR	± 1°
.XXXX	± 0.0005		

DO NOT SCALE DRAWING
BREAK ALL SHARP EDGES AND REMOVE BURRS

THIRD ANGLE PROJECTION

THE SALT RESEARCH GROUP
Berkeley UNIVERSITY OF CALIFORNIA Nuclear Engineering

TITLE: ELECTROCHEMICAL CELL

PROJECT: UCBS-07

DWG NO. 07EG_002 | REV. 0

SCALE 1:2 | SIZE A | SHEET 7 of 7

6 5 4 3 2 1
